# Photo‐Accelerated Synthesis of Oligo(triazole amide)s

**DOI:** 10.1002/marc.202400759

**Published:** 2024-11-13

**Authors:** Alexandros Petropoulos, Laurence Charles, Jean‐Michel Becht, Michael Schmitt, Jacques Lalevée, Jean‐François Lutz

**Affiliations:** ^1^ CNRS UMR 7006, ISIS 8 allée Gaspard Monge Université de Strasbourg Strasbourg 67000 France; ^2^ CNRS UMR 7361, IS2M 15 rue Jean Starcky Université de Haute‐Alsace Mulhouse 68100 France; ^3^ CNRS UMR 7273, ICR Avenue Escadrille Normandie‐Niemen Marseille 13397 France

**Keywords:** click chemistry, photochemistry, precision polymers, sequence‐controlled polymers, uniform oligomers

## Abstract

A photo‐assisted process is explored for improving the synthesis of oligo(triazole amide)s, which are prepared by solid phase synthesis using a repeated cycle of two reactions: amine‐carboxylic acid coupling and copper‐catalyzed azide‐alkyne cycloaddition (CuAAC). The improvement of the second reaction is investigated herein. A catalytic system involving Cu(II)Cl_2_, *N*,*N*,*N’*,*N*″,*N*″‐pentamethyldiethylenetriamine (PMDETA) and a titanocene photoinitiator is explored for reducing the reaction time of CuAAC. This catalyst is first tested on a model reaction involving phenylacetylene and ethyl azidoacetate in DMSO. The kinetics of these model experiments are monitored by ^1^H NMR in the presence of different concentrations of the photoinitiator. It is found that 30 mol% of photoinitiator leads to quantitative reactions in only 8 min. These conditions are then applied to the solid phase synthesis of oligo(triazole amide)s, performed on a glycine‐loaded Wang resin. The backbone of the oligomers is constructed using 6‐heptynoic acid and 1‐amino‐11‐azido‐3,6,9‐trioxaundecane as submonomers. Due to slow reagent diffusion, the CuAAC step required more time in the solid phase than in solution. Yet, one hour only is necessary to achieve quantitative CuAAC on the resin, which is twice as fast as previously‐reported conditions. Using these optimized conditions, oligo(triazole amide)s of different length are prepared.

## Graphical abstract

A photo‐assisted process is explored to improve the time‐consuming copper‐catalyzed azide‐alkyne cycloaddition step used in the solid‐phase synthesis of oligo(triazole amide)s. Employing a Cu(II)Cl_2_/PMDETA complex and a titanocene photoinitiator, reaction times are significantly reduced. Model reaction and solid‐phase synthesis experiments evidence that the use of 30 mol% of photoinitiator as compared to Cu(II) led to fast and efficient oligo(triazole amide) synthesis.

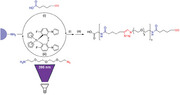

## 1 Introduction

During the last two decades, important efforts have been made for the synthesis of molecularly‐defined oligomers^[^ ^1^ ^]^ with precisely controlled monomer sequence,^[^ ^2^ ^]^ chain length,^[^ ^3^ ^]^ tacticity^[^ ^4^ ^]^ and folding.^[^ ^5^ ^]^ Besides their usefulness in established areas such as drug delivery, antimicrobial surfaces, adhesion and additives,^[^ ^3^, ^6^, ^7^, ^8^ ^]^ these “precision” polymers have also opened up previously unknown applications for synthetic polymers such as data storage,^[^ ^9^, ^10^, ^11^, ^12^, ^13^ ^]^ anti‐counterfeiting barcodes^[^ ^14^, ^15^ ^]^ and cryptography.^[^ ^16^, ^17^ ^]^ Yet, in most cases, uniform oligomers are obtained via multistep growth “polymerizations”.^[^ ^18^ ^]^ In other words, they are not obtained through a simple one pot process but through the repetition of coupling steps, which can be performed on a solid‐support, on a soluble support or in solution. Therefore, their synthesis remains time‐consuming, pricy and limited at best to multi‐kilos scale.^[^ ^19^ ^]^

Among the various families of precision oligomers that have been reported,^[^ ^19^ ^]^ oligo(triazole amide)s^[^ ^20^ ^]^ have been proven to be useful for different applications. Their monomer sequences can be finely controlled using different types of building blocks with main‐chain or side‐chain functional groups. Therefore, oligo(triazole amide)s can store information at the molecular level.^[^ ^10^ ^]^ The first proof‐of‐principle of a non‐biological macromolecule allowing digital storage was obtained with these polymers.^[^ ^21^, ^22^ ^]^ Furthermore, their monomer sequences can be decoded by mass spectrometry.^[^ ^23^ ^]^ Since oligo(triazole amide)s contain amide and triazole groups that are peptide isosteres,^[^ ^24^, ^25^ ^]^ they can also be derived into interesting peptidomimetics and xeno nucleic acids.^[^ ^26^ ^]^ For example, the synthesis of peptide triazole nucleic acids (PTzNA) has been reported.^[^ ^27^ ^]^

**Scheme 1:**
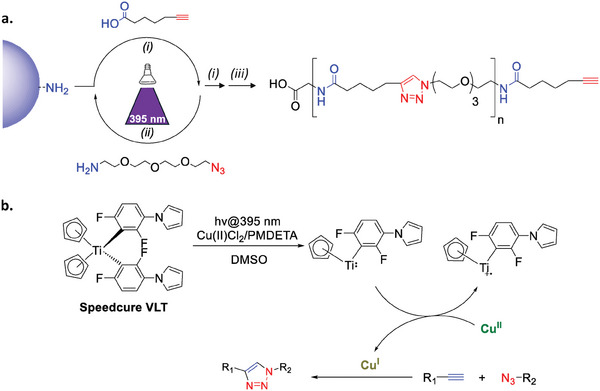
a) General strategy for the iterative solid‐phase synthesis of oligo(triazole amide)s. The blue hemisphere represents a cross‐linked polystyrene solid support. Coupling steps (i) and (ii) are repeated n times to achieve an oligomer of size n. In order to prepare alkyne‐terminated oligomers, an additional step (i) is performed before cleavage from the solid‐support (iii). Experimental conditions: i) HATU, DIPEA, 2,6‐lutidine, DMF, ii) Cu(II)Cl_2_, PMDETA, Speedcure VLT, hv @ 395 nm, DMSO, iii) TFA, DCM. (b) Proposed mechanism of the photoinduced synthesis of triazoles in the presence of a titanocene photoinitiator (Speedcure VLT).

Oligo(triazole amide)s are synthesized via a protecting‐group free iterative process of the AB + CD type, as defined in a recent overview (**Scheme** **1**a).^[^ ^28^ ^]^ This strategy involves two types of building blocks: a submonomer AB containing an acid (reactive group A) and a terminal alkyne (reactive group B) and a submonomer CD containing an azide (reactive group C) and a primary amine (reactive group D). A repetitive cycle involving two successive orthogonal reactions, namely amidification (*step i*) and copper‐catalyzed azide‐alkyne cycloaddition (CuAAC) (*step ii*),^[^ ^29^, ^30^ ^]^ is used to build the triazole‐amide backbone of these polymers. Although orthogonal and useful, this chemistry remains rather slow compared to other multistep processes that have been reported.^[^ ^11^, ^31^ ^]^ The reasons for this are chemical (i.e., reactivity of the selected reacting groups), physico‐chemical (e.g., diffusion of the reactants in the mesh when a solid‐support is used) and technical (i.e., purification times after each repeated step). For instance, CuAAC is a time‐consuming step. Originally, a Cu(I) catalyst was used for these syntheses and overnight reactions were needed to complete the CuAAC *step (ii)*.^[^ ^20^, ^21^ ^]^ In subsequent reports, Cu(II) catalysts were used, which permitted to reduce the coupling time of *step (ii)* to ≈2 h.^[^ ^27^ ^]^ This remains long in comparison with coupling steps in the minute‐range that are used in other iterative syntheses.^[^ ^31^ ^]^ In this context, the aim of the present work was to explore the potential benefits of photo‐assisted CuAAC for the synthesis of oligo(triazole amide)s.^[^ ^32^, ^33^, ^34^, ^35^, ^36^ ^]^ Photons are traceless reagents that can specifically initiate photochemical reactions. In this context, photo‐chemistry could help to accelerate synthesis but may also be employed as useful trigger to achieve sequence control.^[^ ^37^ ^]^ Among all the photo‐CuAAC conditions that have been studied,^[^ ^34^, ^35^, ^36^ ^]^ we selected a near‐UV approach induced by a photoinitiator.^[^ ^38^ ^]^ The use of photoinitiators for the indirect photolysis of the Cu(II) complex is generally preferred over the direct photolysis, in which a ligand‐to‐metal charge transfer takes place, because of its higher rate.^[^ ^35^ ^]^ Additionally, the photoinduced generation of Cu(I) is able to provide spatial and temporal control over the reaction, which is of relevance for materials science applications.^[^ ^36^ ^]^ Moreover, the use of cheap Light Emitting Diodes (LEDs) is also interesting to control the synthesis. This system was tested herein for the solid‐phase synthesis of oligo(triazole amide)s.

## 2 Results and Discussion

### 2.1 Model Reactions in Solution

**Figure 1:**
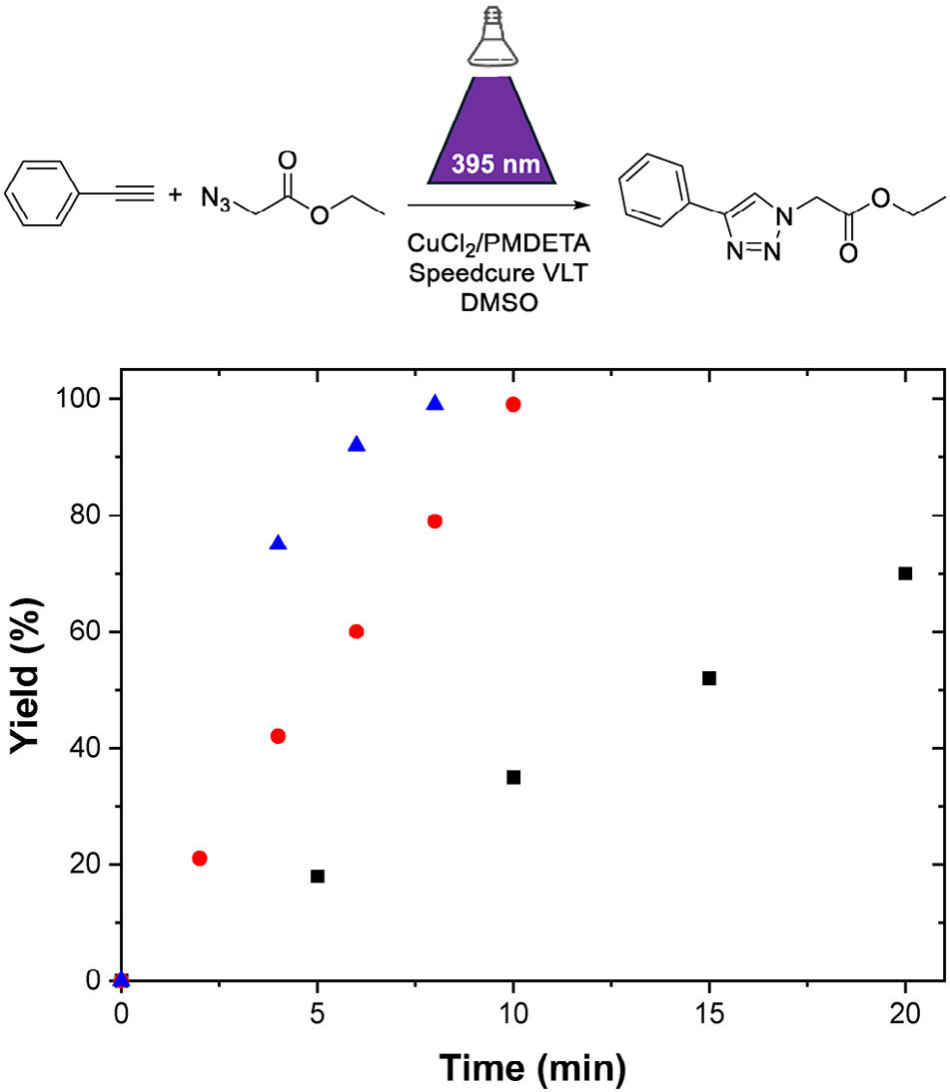
Evolution of reaction kinetics of a model photo‐assisted CuAAC reaction as a function of photoinitiator concentration: 10 (black squares), 20 (red circles) or 30 (blue triangles) mol% of Speedcure VLT. Reactions were carried out under irradiation at 395 nm with an irradiance of 40 mW cm^−2^ at room temperature in DMSO‐d_6_. Initial concentration of phenylacetylene and ethyl azidoacetate were 0.2 mm. Yields were calculated from ^1^H NMR measurements.

At first, model reactions involving phenylacetylene and ethyl azidoacetate (**Figure** **1**) were investigated in order to select suitable CuAAC coupling conditions for the solid‐phase synthesis of oligo(triazole amide)s. The choice of these model substrates enables a clear kinetic study of the reaction through ^1^H NMR spectroscopy (Figure S1, Supporting Information) in contrast to the monomers used in the solid‐phase synthesis of oligo(triazole amide)s. Ideally, photo‐induced CuAAC shall be performed at room temperature in a solvent allowing both a good swelling of the resin^[^ ^39^ ^]^ and the homogenous solubilization of all reactants (i.e., monomer, catalyst/ligand complex and photoinitiator). It is true that there are various solvents in order to perform the CuAAC reaction, with the most common one being a 1:1 mixture of H_2_O/t‐BuOH. However, these two solvents have poor swelling properties on the Wang resin^[^ ^39^ ^]^ used in this work. Hence DMSO was found to be the best compromise and was therefore chosen as the reaction medium. Among the numerous UV and visible light photoinitiators tested over the years to promote the CuAAC reaction, Yagci and coworkers achieved rapid reaction completion while employing a series of different type I or type II photoinitiators.^[^ ^38^ ^]^ The most notable result was obtained using a titanocene photoinitiator, demonstrating faster reaction times and near quantitative yields.^[^ ^38^ ^]^ Titanocene is categorized as a Type I photoinitiator which absorbs photons of appropriate energy after irradiation, and subsequently undergoes a homolytic Ti─C bond cleavage, generating carbon and titanium‐centered radicals responsible for the reduction of Cu(II) to Cu(I) (Scheme 1b).^[^ ^36^, ^40^ ^]^ The efficiency of titanocene and its derivatives in radical generation after irradiation has led to their wide use in photopolymerization related studies.^[^ ^41^, ^42^, ^43^, ^44^ ^]^ To verify these findings, two substrates namely phenylacetylene and ethyl azidoacetate were selected as the model click components, and photoinduced CuAAC reactions were conducted using Speedcure VLT (i.e., fluorinated derivative of titanocene) as the photoinitiator with a Cu(II)Cl_2_/PMDETA complex as the copper source. Photo‐assisted reactions were carried out in DMSO under irradiation at 395 nm(*40* *mW cm^−2^*). Speedcure VLT has a significant absorption maximum at 395 nm, ensuring the efficient generation of free radicals.

A control experiment was first conducted in the absence of irradiation but under the same experimental conditions (models reactants, Cu(II)Cl_2_/PMDETA complex and Speedcure VLT). As expected, no reaction occurred, even after a prolonged period (Figure S2, Supporting Information), highlighting the necessity of light irradiation for the promotion of the CuAAC reaction. Figure 1 shows the evolution of the photoinduced CuAAC reaction kinetics as a function of Speedcure VLT concentration. All reactions were performed in DMSO‐*d_6_* and the kinetics were followed through ^1^H NMR spectroscopy. In the work of Yagci and coworkers a yield of 89% for the reaction in solution was achieved in 10 min while using 10% mol of the photoinitiator,^[^ ^38^ ^]^ which is not in agreement with this work, probably because the efficiency of the CuAAC reaction also depends on the molecular structures of the chosen reactants. After employing higher photoinitiator concentrations under identical experimental conditions, quantitative yields were successfully reached in shorter times. Specifically, a yield of 99% was achieved in 8 min when 30% mol of Speedcure VLT was employed, paving the path for application in the solid‐phase synthesis of oligo(triazole amide)s.

### 2.2 Oligomer Synthesis on Solid Phase

Oligo(triazole amide)s of different length were synthesized using 6‐heptynoic acid (AB submonomer) and 1‐amino‐11‐azido‐3,6,9‐trioxaundecane (CD submonomer) as building blocks. As shown in Scheme 1a, these two submonomers were linked to each other on a solid support through successive amidification (step *(i)*) and photoinduced CuAAC (step *(ii)*) reactions, affording amide and triazole linkages respectively. All oligomers were prepared on a commercial glycine‐loaded Wang resin. In line with previous experiments, a control experiment was conducted in the absence of light and CuAAC did not occur. Step *(i)* involving function A (carboxylic acid) of building block AB and function D (amine) of building block CD was carried out following a previously published protocol.^[^ ^27^ ^]^ Step *(ii)* was performed in DMSO using the same conditions (30 mol% Speedcure VLT) as for the model reaction experiments described above. However, a longer reaction time was selected for performing step *(ii)* on a solid‐support. Indeed, it is well‐known that solid‐phase reactions usually require longer times than their liquid‐phase counterparts due to the limited diffusion of the reactants in the mesh of the solid‐support. Furthermore, in the present case, the yield of the photo‐assisted reaction might also depend on the penetration of light in the resin microbeads. Therefore, preliminary tests were performed in order to determine the optimal LED irradiation duration for step *(ii)*. An oligomer consisting of five building blocks was synthesized with the duration of each step *(ii)* being kept at 40 min. After cleaving the oligomer from the solid‐support and conducting MS analysis, it was found that a non‐negligible quantity of the 3‐building block oligomer was present in the sample, indicating that the CuAAC reaction had not reached completion. The optimal irradiation duration was finally found to be 1 h (instead of 8 min for the model reaction performed in solution). All oligomers were synthesized by performing a given number of iterative cycles, followed by a terminal step *(i)* in order to prepare alkyne‐terminated oligomers. Afterward, the oligomers were cleaved from the solid‐support (step *(iii)* in Scheme 1a) and analyzed by electrospray ionization high‐resolution mass spectrometry (ESI‐HRMS) and NMR (Figure S3, Supporting Information). **Table** **1** shows the molecular structure of the different oligomers prepared in this work.

**Table 1:** Characterization of the different oligo(triazole amide)s synthesized in this work.

	Oligomer structure^a^ ^)^	*m/z*_th_	*m/z*_exp_	Yield [%]
**P1**	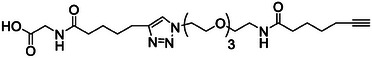	510.2922	510.2920^b^ ^)^	98
**P2**	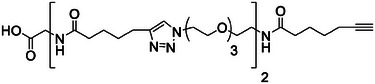	418.7475	418.7481^c^ ^)^	98
**P3**	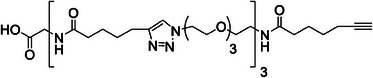	581.8452	581.8457^c^ ^)^	93

^a)^ The listed yields include the whole distribution of the oligomer sample;

^b)^ Detected as [M+H]^+^;

^c)^ Detected as [M+2H]^2+^.

**Figure 2:**
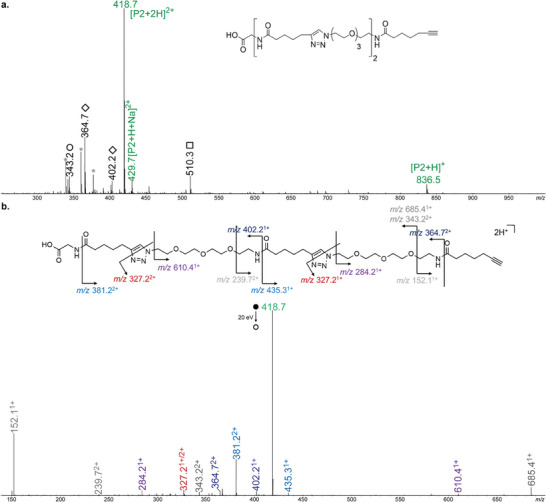
a) Positive mode ESI‐MS spectrum of oligomer **P2**. Grey stars designate peaks from chemical noise. Circles, diamonds, and squares indicate truncated chains with ‐OH, ‐NH_2_ or alkyne ω‐end groups, respectively. b) MS/MS of oligomer **P2** obtained by collision‐induced dissociation of the [**P2** + 2H]^2+^ precursor ion at *m/z* 418.7.

In all cases, the targeted structures were obtained. **Figure** **2** shows the ESI‐MS and MS/MS spectra obtained for oligomer **P2**. The targeted oligomer was clearly identified, even though small peaks due to other species can also be detected. The majority of these oligomeric species are not related to photo‐assisted step *(ii)*. Due to the presence of OH‐terminated impurities in the commercial batch of 1‐amino‐11‐azido‐3,6,9‐trioxaundecane, some OH‐terminated sequences were detected (indicated by circles in Figure 2a). In addition, NH_2_‐terminated sequences can be observed due to incomplete step *(i)* (indicated by in Figure 2a). A small fraction of shorter chains with terminated alkynes (indicated by squares in Figure 2a) was also observed. Comparable results were obtained for longer oligomers such as **P3** (Figure S4, Supporting Information).

## 3 Conclusion

In summary, a photo‐assisted protocol was investigated for the synthesis of molecularly‐defined oligo(triazole amide)s. In particular, safe and cheap light irradiation was explored to improve CuAAC, which is a time‐limiting step for the preparation of these oligomers. At first, a model reaction in solution was explored in order to find experimental conditions that are efficient and suitable for solid‐phase iterative synthesis. A Cu(II)Cl_2_/PMDETA complex in conjunction with a titanocene photoinitiator was selected. It was found that in presence of 30 mol% of the photoinitiator, the model reaction proceeds quantitatively in ≈8 min. Therefore, these conditions were selected for solid‐phase synthesis and applied for the preparation of oligo(triazole amide)s of different sizes. All oligomers were obtained in high yields and exhibited a uniform molecular structure, as evidenced by mass spectrometry analysis. Perhaps more importantly, the CuAAC step could be completed in only one hour on the resin, which reduces by half the coupling time as compared to previously reported protocols.^[^ ^27^ ^]^ These results indicate that light‐assisted synthesis is a promising strategy for improving the preparation of oligomers by multistep growth “polymerizations”. Indeed, light‐assisted protocols may potentially be applied to accelerate synthesis of a wide variety of sequence‐defined oligomers.^[^ ^19^ ^]^ Ultimately, such strategies may also lead to efficient iterative syntheses that are fully controlled by light.

## Conflict of Interest

The authors declare no conflict of interest.

## Supporting information



Supporting Information

## Data Availability

The data that support the findings of this study are available from the corresponding author upon reasonable request.
