# Supplementary material for: Photo‐Accelerated Synthesis of Oligo(triazole amide)s
Source: Macromol Rapid Commun. 2024 Nov 13;46(4):2400759. doi: 10.1002/marc.202400759 (PMC11841660; doi:10.1002/marc.202400759)
Supplement: Supplementary file 1 — Supporting Information [file MARC-46-2400759-s001.pdf]

Macromolecular Rapid Communications

Supporting Information

for *Macromol. Rapid Commun.*, DOI 10.1002/marc.202400759

Photo-Accelerated Synthesis of Oligo(triazole amide)s

Alexandros Petropoulos, Laurence Charles, Jean-Michel Becht, Michael Schmitt, Jacques Lalevée and Jean-François Lutz**

Supporting Information

Photo-accelerated Synthesis of Oligo(triazole amide)s

Alexandros Petropoulos, Laurence Charles, Jean-Michel Becht, Michael Schmitt, Jacques Lalevée and Jean-François Lutz**

A. Experimental section

A.1. Materials and methods

Phenylacetylene (Alfa Aesar, 98%), ethyl azidoacetate (TCI, >95%), bis(η5-cyclopentadienyl)-bis(2,6-difluoro-3-[pyrrol-1-yl]-phenyl)titanium (Speedcure VLT, Arkema), N,N,N',N'',N''-pentamethyldiethylenetriamine (PMDETA, TCI, >99%), Fmoc-Gly Wang resin (Novabiochem, 99%), 2-(1H-7-azabenzotriazol-1-yl)-1,1,3,3-tetramethyluronium hexafluorphosphate (HATU, Iris Biotech, 99%), N,N-diisopropylethylamine (DIPEA, TCI, 99%), 2,6-lutidine (Alfa Aesar, 98%), 6-heptynoic acid (Alfa Aesar, 95%), 1-amino-11-azido-3,6,9-trioxaundecane (ABCR, 95%), trifluoroacetic acid (TFA, Alfa Aesar, 99%), dichloromethane (DCM, Sigma-Aldrich, 99.9%, stabilized with amylene), N,N-dimethylformamide anhydrous (DMF anhydrous, Sigma-Aldrich, 99.8%), N,N-dimethylformamide (DMF, Sigma-Aldrich, 99%) were used as received. Copper(II) chloride dihydrate ($CuCl_2$ $2H_2O$, Sigma-Aldrich, 99%) was dried in a vacuum oven in order to get rid of the water. The successful drying can be observed by the change from the blue-green color of the $CuCl_2$ $2H_2O$ complex to brown of the desired $CuCl_2$.

A.2. Light-induced copper(I)-catalyzed model click reaction

In a small glass vial containing Cu(II)Cl_2 (2.68 mg, 0.02 mM), PMDETA (4.4 µL, 0.02 mM) and the selected Speedcure VLT photoinitiator (3 different concentrations, 0.02 mM, 0.04 mM and 0.06 mM corresponding to 10%, 20% and 30% mol of the reactants, respectively), 1 mL of DMSO-d_6 and ethyl azidoacetate (23.1 µL, 0.2 mM) were added. After 2 min, the phenyl acetylene was finally added (26 µL, 0.2 mM), the vial was sealed with a rubber septum and degassed by bubbling with for approximately 5 minutes. The glass vial was then irradiated by a ThorLabs LED lamp emitting light at 395 nm with a power density of 40 mW cm^{-2}. ^{1}H-NMR spectra were recorded at different time intervals during the reaction with the help of aliquots, with each measurement involving 16 scans. Final conversions were determined through integration of the signal from a proton in one of the starting compounds and the corresponding proton in the targeted product (*e.g.*, protons **a** and **b** in Figure S1). ^{1}H NMR (400 MHz, DMSO-

d_6, δ, ppm): 1.24 (t, 3H, -CH_2-C$\underline{H}_3$), 4.21 (q, 2H, -O-C$\underline{H}_2$-CH_3), 5.46 (s, 2H, -N-C$\underline{H}_2$-CO-), 7.35 (t, 1H, aromatic), 7.46 (t, 2H, aromatic), 7.87 (d, 2H, aromatic), 8.58 (s, 1H, -N-C$\underline{H}$=C-). The protons that are not reported but can however be seen through the peaks observed in Figure S1 are attributed to degradation products coming from the cleavage of the photoinitiator (Speedcure VLT) and also from PMDETA.

A.3. Activation of Fmoc-Gly-Wang resin

0.05 g (loading = 0.36 mmol/g, 1eq.) of Fmoc-Gly-Wang resin was used as a solid support and placed in a 10 mL SPE tube. The resin beads were firstly swollen by shaking in DCM for 10 min. Next, the Fmoc group was removed by treatment with piperidine in DMF (20:80 v:v) for 20 min. The deprotection step was repeated to ensure complete removal of the Fmoc protecting groups from the resin beads.

B.4. Solid-phase synthesis of oligo(triazole amide)s.

The following sub-sections describe the coupling of submonomers AB and CD to the resin. Steps *(i)* and *(ii)* are repeated a certain number of times until an oligomer of the desired length is reached. In order to prepare alkyne-terminated oligomers, an additional step *(i)* is performed before cleavage from the solid-support.

B.4.1. Coupling of the AB submonomer via step (i).[1] Three solutions were prepared: a solution of the coupling agent, HATU (0.5 M in anhydrous DMF), a solution of bases (DIPEA and 2,6-lutidine in anhydrous DMF, 1.2 and 1.8 M respectively) and a solution of monomer (0.2 M in anhydrous DMF). The resin was firstly conditioned with anhydrous DMF and then the SPE tube was emptied without completely drying the resin. In a small glass vial, a mixture was prepared composed of the monomer solution (4 eq.), the coupling agent solution (3.5 eq) and the bases solution (DIPEA 4 eq. and 2,6-lutidine 6 eq.). The mixture was let in the glass vial for 2 min in order to activate the carboxylic acid of the monomer. It was then collected with a Pasteur pipette and deposed on the resin inside the SPE tube for 30 min. The SPE tube was then emptied and a fresh mixture was added for another 30 min. The SPE tube was emptied once again and the resin was washed with solvents (DMF 4x, DCM 4x, DMF 2x, DCM 2x).

B.4.2. Coupling of the CD submonomer via step (ii). The resin was firstly conditioned with DMSO, dried and transferred to a 5 mL round bottom flask. Then, the copper source (Cu(II)Cl_2, 3 eq.), the ligand (PMDETA, 6 eq.), and the monomer (8.5 eq.) were added. A rubber septum was then placed to close the round bottom flask and the mixture was purged with argon to

achieve an oxygen-free atmosphere. 1.5 mL of solvent (DMSO) was injected in the round bottom flask under argon. The reaction mixture was then irradiated with a ThorLabs LED lamp emitting light at 395 nm with an of 40 mW cm^{-2} for 1h under vigorous stirring. The solution was transferred to the SPE tube and finally drained. Then the resin was washed a first time with solvents (DMSO 4x, DCM 4x, DMSO 2x, DCM 2x), with a saturated solution of EDTA in a mixture of water and DMF (1:1) during 10 min, in order to get rid of the last traces of copper, and with solvents again (DMSO 4x, DCM 4x, DMSO 2x, DCM 2x).

B.4.3. Procedure for cleavage via step (iii). Cleavage of the oligo(triazole amide)s from the resin was performed in a TFA/DCM solution (1:1 v:v) for 1.5 h inside the SPE tube. After cleavage, the solution was filtered through the frit into a 10 mL glass vial. The resin was washed three times with DCM in order to recover all the amount of the desired oligomer. The mixture of TFA/DCM in the glass vial was then let to evaporate and was finally dried *in vacuo*.

B. Measurements and analyses

B.1. Nuclear Magnetic Resonance (NMR)

^{1}H NMR spectra were recorded in DMSO-d_6 using a Bruker Avance spectrometer equipped with Ultrashield magnets and operating at 400 MHz for ^{1}H NMR. The ^{1}H NMR spectra were referenced to the residual proton impurities at δ H 2.50 ppm (DMSO-d_6).

B.2. Mass Spectrometry.

High resolution MS and MS/MS experiments were performed using a QqTOF mass spectrometer (ZenoTOF 7600, SCIEX, Concord, ON, Canada) equipped with an electrospray ionization (ESI) source operated at room temperature in the positive ion mode.[2] The spray voltage was set to +5.5 kV and the declustering potential was +75 V. Air was used as the nebulizing gas (20 psi) while nitrogen was used as the curtain gas (35 psi) as well as the collision gas (7 psi) in MS/MS experiments. The Zeno trap pulsing was activated, using an accumulation time of 0.1 s. Instrument control, data acquisition and data processing were achieved using SCIEX OS 3.4 provided by SCIEX. A few mg of each polymer was dissolved in methanol and then diluted (1/100) in a methanolic solution of ammonium acetate (3 mM) prior to injection in the ESI source at 10 μL/min using a syringe pump.

C. Additional Figures

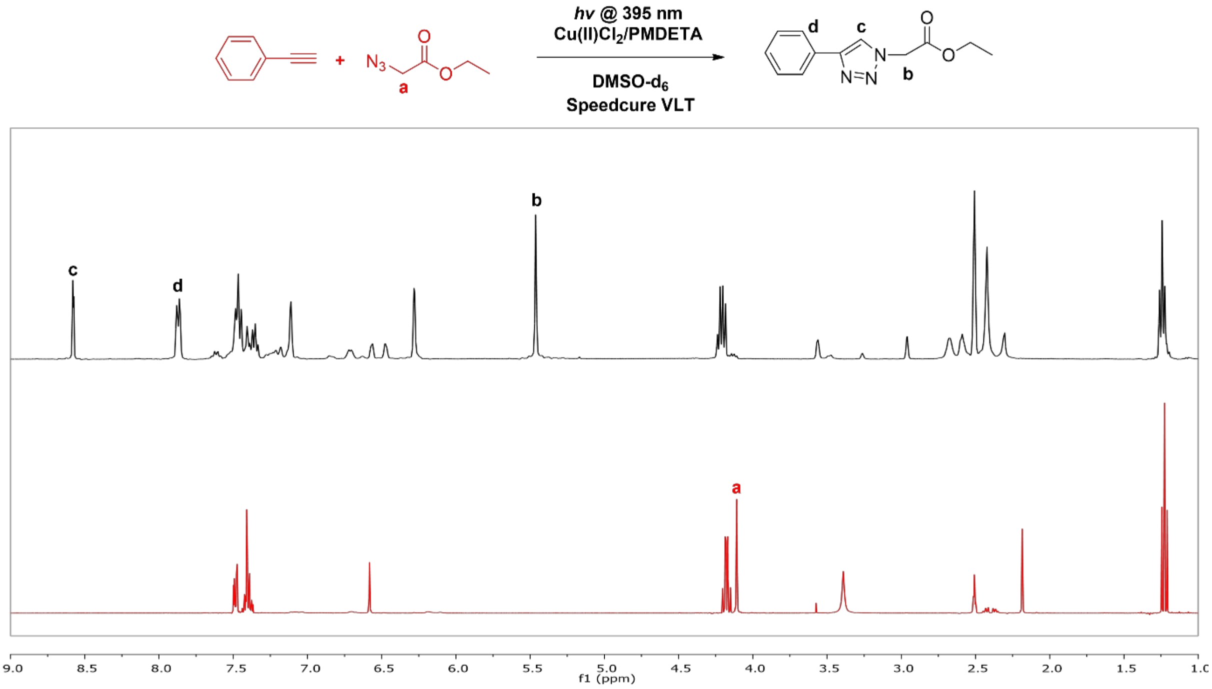

Figure S1. ^{1}H NMR spectra of the starting materials mixture (red) and the targeted product (black) by the photoinduced CuAAC click reaction in DMSO-*d*$_6$.

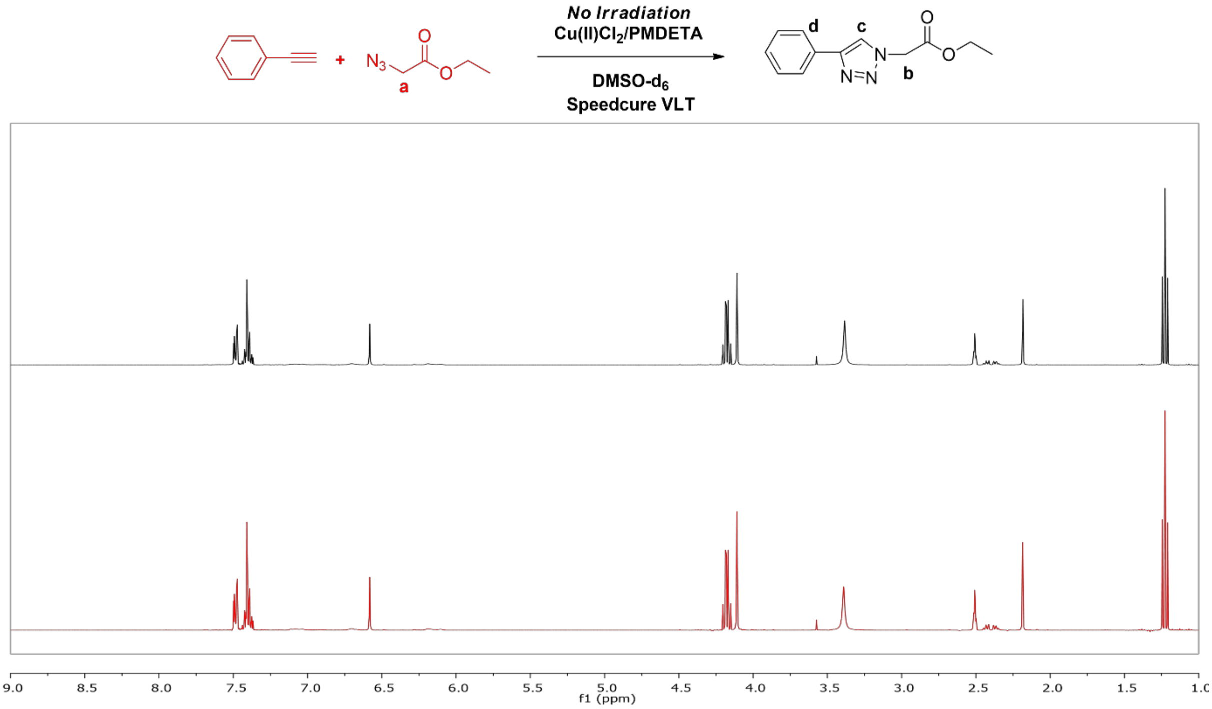

Figure S2. ^{1}H NMR spectra of the reaction mixture before (red) and after 30 minutes (black) in the absence of light irradiation in DMSO-*d*$_6$.

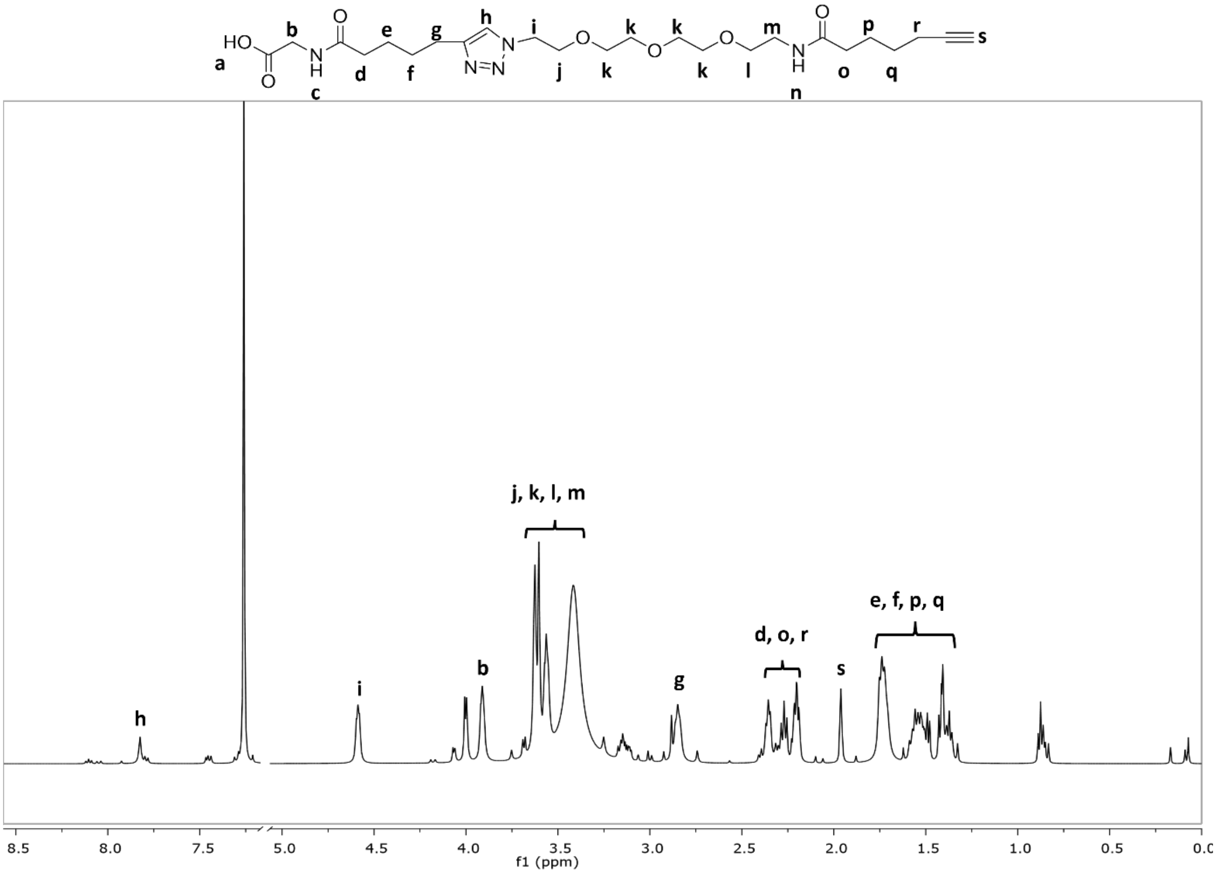

Figure S3. ^{1}H NMR spectrum of **P1** recorded in $CDCl_3$.

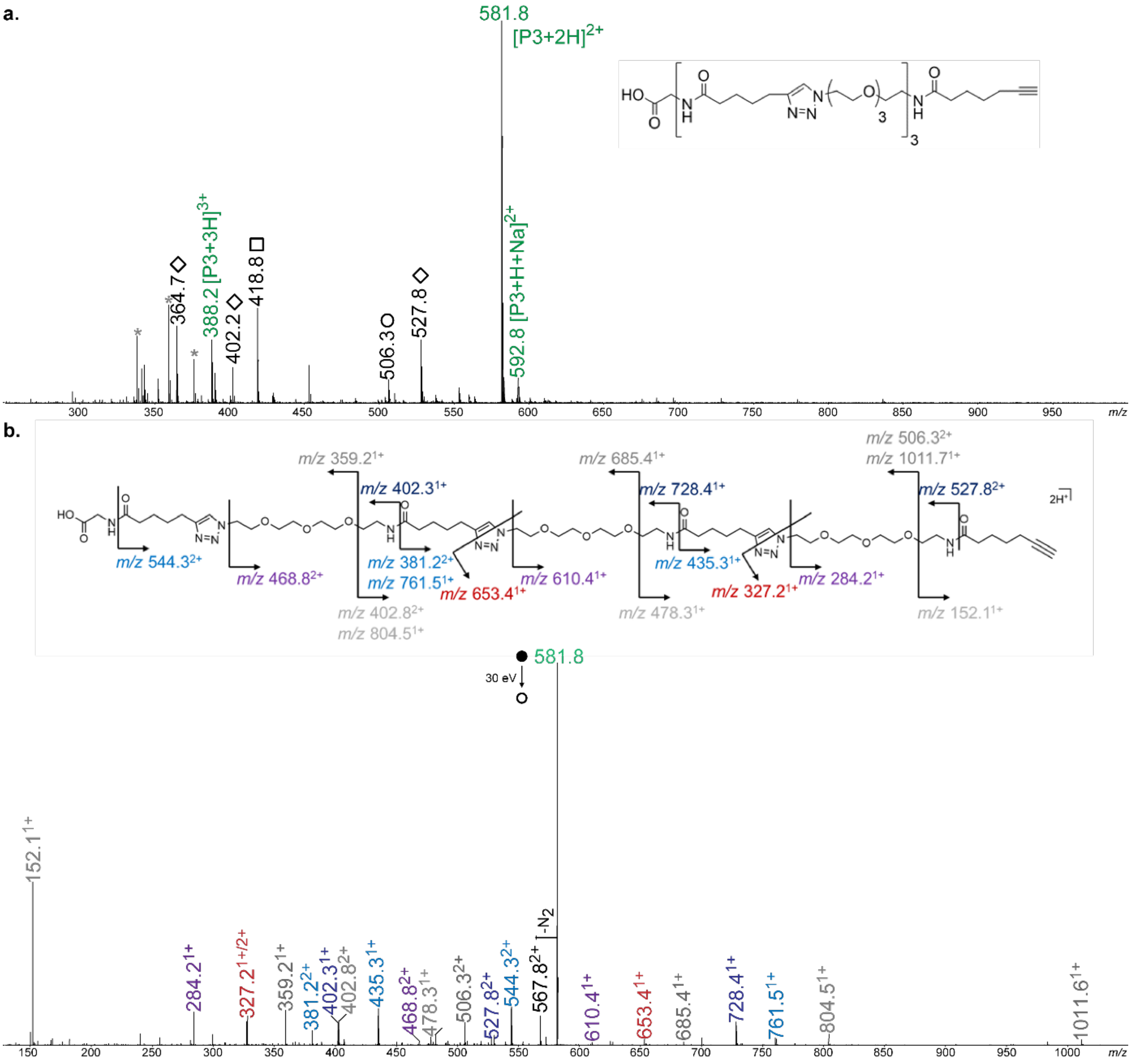

Figure S4. (**a**) Positive mode ESI-MS spectrum of oligomer **P3**. Grey stars designate peaks from chemical noise. Circles, diamonds and squares indicate truncated chains with -OH, -NH_2 or alkyne ω-end groups, respectively. (**b**) MS/MS of oligomer **P3** obtained by collision-induced dissociation of the [**P3** + 2H]$^{2+}$ precursor ion at *m/z* 581.8.

D. References

[1] G. Fiers, D. Chouikhi, L. Oswald, A. Al Ouahabi, D. Chan-Seng, L. Charles, J. F. Lutz, *Chem. Eur. J.* **2016**, *22*, 17945-17948.

[2] J.-A. Amalian, T. T. Trinh, J.-F. Lutz, L. Charles, *Anal. Chem.* **2016**, *88*, 3715-3722.
